# A replication study of JTC bias, genetic liability for psychosis and delusional ideation

**DOI:** 10.1017/S0033291720003578

**Published:** 2022-07

**Authors:** Cécile Henquet, Jim van Os, Lotta K. Pries, Christian Rauschenberg, Philippe Delespaul, Gunter Kenis, Jurjen J. Luykx, Bochao D. Lin, Alexander L. Richards, Berna Akdede, Tolga Binbay, Vesile Altınyazar, Berna Yalınçetin, Güvem Gümüş-Akay, Burçin Cihan, Haldun Soygür, Halis Ulaş, Eylem S. Cankurtaran, Semra U. Kaymak, Marina M. Mihaljevic, Sanja S. Petrovic, Tijana Mirjanic, Miguel Bernardo, Gisela Mezquida, Silvia Amoretti, Julio Bobes, Pilar A. Saiz, Maria P. García-Portilla, Julio Sanjuan, Eduardo J. Aguilar, Jose L. Santos, Estela Jiménez-López, Manuel Arrojo, Angel Carracedo, Gonzalo López, Javier González-Peñas, Mara Parellada, Nadja P. Maric, Cem Atbaşoğlu, Alp Ucok, Köksal Alptekin, Meram C. Saka, Celso Arango, Michael O'Donovan, Bart P.F. Rutten, Sinan Gülöksüz

**Affiliations:** 1 Department of Psychiatry and Neuropsychology, School for Mental Health and Neuroscience, Maastricht University Medical Centre, Maastricht, the Netherlands; 2 Department of Psychiatry, UMC Utrecht Brain Center, University Medical Center Utrecht, Utrecht University, Utrecht, The Netherlands; 3 Department of Psychosis Studies, King's College London, Institute of Psychiatry, London, UK; 4 Department of Public Mental Health, Central Institute of Mental Health, Medical Faculty Mannheim, University of Heidelberg, Mannheim, Germany; 5 Department of Translational Neuroscience, UMC Utrecht Brain Center, University Medical Center Utrecht, Utrecht University, Utrecht, The Netherlands; 6 GGNet Mental Health, Apeldoorn, The Netherlands; 7 MRC Centre for Neuropsychiatric Genetics and Genomics, Division of Psychological Medicine and Clinical Neurosciences, School of Medicine, Cardiff University, Cardiff, UK; 8 Department of Psychiatry, Faculty of Medicine, Dokuz Eylul University, Izmir, Turkey; 9 Department of Psychiatry, Faculty of Medicine, Adnan Menderes University, Aydin, Turkey; 10 Department of Neuroscience, Health Sciences Institute, Dokuz Eylul University, Izmir, Turkey; 11 Department of Physiology, School of Medicine, Ankara University, Ankara, Turkey; 12 Brain Research Center, Ankara University, Ankara, Turkey; 13 Department of Psychology, Middle East Technical University, Ankara, Turkey; 14 Turkish Federation of Schizophrenia Associations, Ankara, Turkey; 15 Department of Psychiatry, Faculty of Medicine, Dokuz Eylul University, Izmir, Turkey (Discharged by statutory degree No: 701 at 8th July of 2018, because of signing “Peace Petition”); 16 Güven Çayyolu Healthcare Campus, Ankara, Turkey; 17 Atatürk Research and Training Hospital Psychiatry Clinic, Ankara, Turkey; 18 Faculty of Medicine, University of Belgrade, Belgrade, Serbia; 19 Clinic for Psychiatry CCS, Belgrade, Serbia; 20 Special Hospital for Psychiatric Disorders Kovin, Kovin, Serbia; 21 Barcelona Clinic Schizophrenia Unit, Neuroscience Institute, Hospital Clinic of Barcelona, University of Barcelona, Barcelona, Spain; 22 Institut d'Investigacions Biomèdiques August Pi I Sunyer, Barcelona, Spain; 23 Biomedical Research Networking Centre in Mental Health (CIBERSAM), Spain; 24 Instituto de Investigación Sanitaria del Principado de Asturias, Oviedo, Spain; 25 Department of Psychiatry, School of Medicine, University of Oviedo, Oviedo, Spain; 26 Mental Health Services of Principado de Asturias, Oviedo, Spain; 27 Department of Psychiatry, Hospital Clínico Universitario de Valencia, School of Medicine, Universidad de Valencia, Valencia, Spain; 28 Department of Psychiatry, Hospital Virgen de la Luz, Cuenca, Spain; 29 Health and Social Research Center, Universidad de Castilla-La Mancha, Cuenca, Spain; 30 Department of Psychiatry, Instituto de Investigación Sanitaria, Complejo Hospitalario Universitario de Santiago de Compostela, Santiago de Compostela, Spain; 31 Grupo de Medicina Genómica, Centro de Investigación Biomédica en Red de Enfermedades Raras (CIBERER) Universidad de Santiago de Compostela, Santiago de Compostela, Spain; 32 Fundación Publica Galega de Medicina Xenómica (SERGAS), IDIS, Santiago de Compostela, Spain; 33 Department of Child and Adolescent Psychiatry, Institute of Psychiatry and Mental Health, Hospital General Universitario Gregorio Marañón, IiSGM, School of Medicine, Universidad Complutense, Madrid, Spain; 34 Institute of Mental Health, Belgrade, Serbia; 35 Department of Psychiatry, School of Medicine, Ankara University, Ankara, Turkey; 36 Department of Psychiatry, Faculty of Medicine, Istanbul University, Istanbul, Turkey; 37 Department of Psychiatry, Yale School of Medicine, New Haven, CT, USA

**Keywords:** Cognition, delusions, family, jumping to conclusions, neuropsychology, psychosis, reasoning

## Abstract

**Background:**

This study attempted to replicate whether a bias in probabilistic reasoning, or ‘jumping to conclusions’(JTC) bias is associated with being a sibling of a patient with schizophrenia spectrum disorder; and if so, whether this association is contingent on subthreshold delusional ideation.

**Methods:**

Data were derived from the EUGEI project, a 25-centre, 15-country effort to study psychosis spectrum disorder. The current analyses included 1261 patients with schizophrenia spectrum disorder, 1282 siblings of patients and 1525 healthy comparison subjects, recruited in Spain (five centres), Turkey (three centres) and Serbia (one centre). The beads task was used to assess JTC bias. Lifetime experience of delusional ideation and hallucinatory experiences was assessed using the Community Assessment of Psychic Experiences. General cognitive abilities were taken into account in the analyses.

**Results:**

JTC bias was positively associated not only with patient status but also with sibling status [adjusted relative risk (aRR) ratio : 4.23 CI 95% 3.46–5.17 for siblings and aRR: 5.07 CI 95% 4.13–6.23 for patients]. The association between JTC bias and sibling status was stronger in those with higher levels of delusional ideation (aRR interaction in siblings: 3.77 CI 95% 1.67–8.51, and in patients: 2.15 CI 95% 0.94–4.92). The association between JTC bias and sibling status was not stronger in those with higher levels of hallucinatory experiences.

**Conclusions:**

These findings replicate earlier findings that JTC bias is associated with familial liability for psychosis and that this is contingent on the degree of delusional ideation but not hallucinations.

## Introduction

Cognitive models of psychosis suggest that dysfunctions in decision-making are involved in the formation and maintenance of positive states of psychosis (Garety & Freeman, 1999). In this process, which has been described as jumping to conclusions (JTC), individuals appraise ambiguous stimuli and come to a conclusion based on limited information, which in turn may result in delusional experience (Freeman & Garety, 2014). Over the past decades, numerous studies have investigated JTC bias in relation to psychosis, including clinical (Garety et al., 2005; So et al., 2012) and non-clinical samples (Freeman, Pugh, & Garety, 2008), and different levels of psychosis expression (subthreshold, clinical) (Broome et al., 2007). Several meta-analyses have been conducted, including many studies, examining the overall strength and specificity of JTC bias in psychosis (Dudley, Taylor, Wickham, & Hutton, 2016; McLean, Mattiske, & Balzan, 2017; So, Siu, Wong, Chan, & Garety, 2016). Meta-analysis has revealed that JTC bias is consistently associated with psychosis and that the strength of the association is considered to be moderate (Dudley et al., 2016; McLean et al., 2017; So et al., 2016). Compared with healthy controls, patients with psychosis use significantly less information in reaching a decision and show a stronger tendency of extreme responding (Dudley et al., 2016). Furthermore, the association between JTC seems to be primarily related to psychotic states, as associations with JTC bias in patients with non-psychotic mental disorders are weaker (McLean et al., 2017) and highly heterogeneous (So et al., 2016). Moreover, the association between JTC appears to be delusion-specific, as people with psychotic illness with a higher level of delusional ideation show greater JTC bias than those with no delusional ideation (Dudley et al., 2016; Ross, McKay, Coltheart, & Langdon, 2015). Although there is some evidence that the association between JTC and psychosis is stronger in individuals with evidence of current symptomatology (Dudley et al., 2016), the question whether JTC bias is a trait or a state characteristic of psychosis remains to be elucidated. Given that healthy participants without psychosis show a degree of JTC bias, albeit to a lesser extent than patients, and that JTC bias is observed in patients without delusions, JTC bias appears to be neither a sufficient nor a necessary cause in the formation of delusions. In addition, the factors that contribute to JTC bias remain to be elucidated. One study investigated familial risk to psychosis as a possible contributing factor to JTC bias (Van Dael et al., 2006). In this study, JTC bias was examined in four groups with different levels of psychosis liability: (i) 40 patients with a history of non-affective psychosis, (ii) first degree relatives of patients with a history of non-affective psychosis, (iii) participants scoring high (>75^th^ percentile) on the positive dimensions of psychosis proneness (measured with the Community Assessment of Psychic Experiences, CAPE) (Konings, Bak, Hanssen, van Os, & Krabbendam, 2006) – see under ‘Instruments’ for a description of the questionnaire), (iv) healthy controls scoring in the average range (40^th^ – 60^th^ percentile on the CAPE). JTC was assessed using the beads tasks and JTC bias was defined as requesting only a single bead before deciding (see under ‘Instruments’ for a description of the task). A significant dose-response relationship was found between the level of psychosis liability (group) and JTC, and the association was conditional on the presence of delusional ideation (a dichotomized score on the delusion subscale of the Present State Examination, PSE) (Wing, Cooper, & Sartorius, 1974). These findings suggest that JTC is in part a trait associated with liability for psychosis, but also a state-related phenotype, given its specific association with delusional ideation. The current study aimed to replicate the findings from the study by Van Dael et al. (2006), now using a much larger study sample. The aim of this study was to (i) investigate the association between JTC bias and familial liability for psychosis, using the status of non-affected sibling of a patient with the psychotic disorder as a marker of familial risk, and (ii) investigate whether any association with sibling status was specific for subthreshold delusional but not hallucinatory states.

## Materials and methods

### Participants

The EUGEI project is a 25-centre, 15-country, EU-funded collaborative network studying the impact of genetic and environmental factors on the onset, course and neurobiology of psychosis spectrum disorder (van Os, Guloksuz, Vijn, Hafkenscheid, & Delespaul, 2019). WorkPackage 6, ‘Vulnerability and Severity’, focused on the psychometric expression of genetic and environmental liability in the siblings of patients, who are at higher than average genetic and environmental risk compared to well healthy comparison participants. The sample in WorkPackage 6 was collected in Spain (five centres), Turkey (three centres), and Serbia (one centre) and consisted of 1525 healthy comparison participants, 1261 patients with a diagnosis of schizophrenia spectrum disorder (average duration of illness since the age of the first contact with mental health services: 9.9 years) and 1282 siblings of these patients without a diagnosis of schizophrenia spectrum disorder. Healthy comparison participants without a diagnosis of schizophrenia spectrum disorder and without a sibling diagnosed with schizophrenia spectrum disorder were recruited from the general population. For a full description of the study see Guloksuz et al. (2019) and van Os et al. (2019). Exclusion criteria for all participants were diagnosis of psychotic disorder due to another medical condition, history of head injury with loss of consciousness and intelligence quotient <70. To achieve high quality and homogeneity in clinical, experimental, and environmental assessments, standardized instruments were administered by psychiatrists, psychologists, or trained research assistants who completed mandatory on-site training sessions and online training modules including interactive interview videos and self-assessment tools (European Network of National Networks studying Gene-Environment Interactions in Schizophrenia et al., 2014). Both on-site and online training sessions were repeated annually to maintain high inter-rater reliability throughout the study enrolment period (for details see: https://cordis.europa.eu/result/rcn/175696_en.html). The EU-GEI project was approved by the Medical Ethics Committees of all participating sites and conducted in accordance with the Declaration of Helsinki (World Medical Association, 2013). All respondents provided written informed consent and, in the case of minors, such consent was also obtained from parents or legal guardian.

### Instruments

#### Beads task

The most widely used task to assess JTC is the beads task in which participants are shown two jars containing green and red beads with equal but opposite ratios (85 red and 15 green beads and vice versa) (Huq, Garety, & Hemsley, 1988; Phillips & Edwards, 1966). Participants are informed of the proportions, and the jars are removed from view. One of the jars then is chosen, hidden from view, and a bead is drawn from it and shown on the screen to the participant. Beads are sequentially drawn and always replaced. Although the participants are told that beads are being selected randomly, the sequence of colours is predetermined according to the ratio of the two colours. Participants are then asked to decide whether the beads are drawn from the mainly green or the mainly red jar. In the condition used in this study, participants were free to determine how many beads were drawn, and the trial was terminated once participants confirmed they were certain about their choice. In this study, a computerized version of the beads task by Phillips & Edwards (1966) was used. In order to assess jumping to conclusions, JTC bias was defined as deciding after only a single bead was drawn (see under ‘analyses’).

#### General intelligence

Cognitive ability was estimated based on a short version of the WAIS-III short form: the Digit Symbol Coding subtest, uneven items of the Arithmetic subtest, uneven items of the Block Design subtest, every third item of the Information subtest (Blyler, Gold, Iannone, & Buchanan, 2000; Velthorst et al., 2013; Wechsler, 1997). Conforming to our previous analyses we calculated, for each test, the *Z*-score, separately for each country and sex (van Os et al., 2019). The cognition score was the mean of the *Z*-scores of the different tests, expressed as a T-score (cognition score shifted and scaled to have a mean of 50 and a standard deviation of 10), with higher scores representing better performance. The measure will be referred hereafter as *‘cognition score’*.

#### Lifetime experience of positive psychotic experiences

The CAPE (www.cape42.homestead.com) was developed to rate self-reports of 42 items, of which 20 on lifetime psychotic experiences (Konings et al., 2006). Items are scored on a 4-point scale rating frequency of the experience.

## Analyses

Statistical analyses were carried out with STATA version 15 (StataCorp, 2017). A 3-level group variable was constructed reflecting the hypothesized order of psychosis risk strata, with controls (coded 0), siblings (coded 1) and patients (coded 2). The number of beads requested yielded a continuous variable within a range from 0 to 20. Based on the earlier work by Van Dael et al. (2006) on the association between psychosis proneness and JTC bias, a variable was *a priori* constructed indicating whether there was a JTC bias, defined as requesting only a single bead before deciding (hereafter ‘JTC bias’) *v.* two beads or more before deciding. CAPE dimensions of the frequency of positive experiences (20 items) were included representing the person's positive psychotic experiences over the lifetime, hereafter ‘CAPE total positive score’. In line with earlier research investigating underlying dimensions of psychotic experiences measured with the CAPE (Wigman et al., 2011) lifetime presence of delusional experiences (based on 17 CAPE-items) was defined as the mean of all CAPE items on the delusions, paranoia, grandiosity and paranormal beliefs dimensions, hereafter ‘CAPE delusions’. ‘CAPE auditory hallucinations’ (based on 2 CAPE items) was calculated as the mean of CAPE items assessing lifetime auditory hallucinatory experiences. Likewise, ‘CAPE visual hallucinations’ was calculated (based on one item). Socio-demographic characteristics age (continuous variable), sex (0 = male, 1 = female), educational level (six levels, 1 = compulsory education no qualifications, 2 = compulsory education with qualifications, 3 = first level of non-compulsory education, 4 = vocational education, 5 = university undergraduate, 6 = university postgraduate) and cognition score were compared across groups using chi-square test (sex and group) and linear regression analyses (with group as independent variable and age, education level and cognition score, respectively, as dependent variable).

### JTC bias and psychosis risk

Psychosis risk as a function of JTC bias was examined using multinomial logistic regression analysis. Effect sizes were expressed as relative risk ratio (RR) with their 95% confidence intervals. Post-estimation Wald test was used to compare relative risk estimates for siblings and patients. The following *a priori* selected confounders were included in the logistic regression model: age, sex, level of education (continuous variable) and cognition score. To adjust for any effects of country, country (three categories with Turkey as reference category) was entered in the model as well. In addition, standard errors were corrected for clustering of siblings and patients within the same family, using the Stata ‘cluster’ option.

### JTC bias and delusions, auditory hallucinations and visual hallucinations

To investigate whether the association between JTC bias and psychosis risk was stronger with higher levels of CAPE delusions, the two-way interaction JTC bias × CAPE delusions was fitted in the multinomial logistic regression analysis. To test for specificity of any moderating effect of JTC and delusions on the outcome measure, the model was separately tested with the interaction JTC bias × CAPE auditory hallucinations and JTC × CAPE visual hallucinations.

## Results

### Sample

In total, 4068 participants were included, of which 1865 (46%) were female, with 1261 patients with a psychotic disorder (31% of the total sample), 1282 first degree siblings of the patients (32%), and 1525 controls (37%) (Table 1). There were no large or significant differences in age across the groups: patients, relatives and controls (*β* = −0.10, 95% CI = −0.48 to 0.28, *p* = 0.612). There were significant sex differences between the three groups (χ^2^ = 12287, df = 2, *p* < 0.001). Educational level was also significantly associated with the group (*β* = −2.45, 95% CI = −2.75 to −2.15, *p* < 0.001) and cognition score (*β* = −0.19, 95% CI = −0.24 to −0.14, *p* < 0.001) (Table 2).

**Table 1.** Sample demographics by group and country

Group			Educational level^a^						
	Age		Sex	Percentage per level						Number
	Mean	S.D.	Percentage female	Level 1	Level 2	Level 3	Level 4	Level 5	Level 6	*n*
Group										
Controls	33.9	10.4	49%	2%	20%	17%	29%	25%	7%	1525
Siblings	34.1	9.4	54%	3%	17%	18%	26%	24%	12%	1282
Patients	33.7	8.6	33%	5%	23%	27%	29%	12%	4%	1261
Country										
Turkey	33.5	9.9	48	2%	21%	16%	32%	26%	3%	2499
Spain	35.1	9.1	42	6%	20%	25%	22%	11%	16%	1415
Serbia	29.3	6.5	52	6%	2%	51%	17%	18%	6%	154
Total	33.9	9.6	46	3%	20%	21%	28%	21%	7%	4068

*Note*: s.d. = standard deviation, *n* = number of observations.

a Educational level: Level 1 = compulsory education no qualifications, Level 2 = compulsory education with qualifications, Level 3 = first level of non-compulsory education, Level 4 = vocational education, Level 5 = university undergraduate, Level 6 = university postgraduate.

**Table 2.** Cognitive scores by group and country

	Cognition score		Beads task number drawn		Number
	Mean	s.d.	Mean	s.d.	*n*
Group					
Controls	49.4	6.8	5.7	3.6	1525
Siblings	50.0	7.2	4.3	4.7	1282
Patients	44.3	7.5	2.9	3.3	1261
Total	48.1	7.6	4.5	4.1	4068
Country					
Turkey	48.0	7.6	4.6	4.0	2499
Spain	48.3	7.7	4.4	4.2	1415
Serbia	47.8	7.0	3.7	3.9	154

*Note*: s.d. = standard deviation, *n* = number of observations.

### Association between JTC bias and psychosis risk

Overall, 35% of the participants displayed evidence of JTC bias: 17% of the control group, 43% of the sibling group, and 52% of the patient group (Table 3). Multinomial logistic regression analysis showed a significant association between JTC bias and psychosis risk (Table 4): both sibling status and patient status were associated with JTC bias, with the greater effect size for patient status (unadjusted association: RR = 3.78, 95% CI 3.16–4.51 for the sibling group compared to the control group and RR = 5.42, 95% CI 4.53–6.50 for the patient group compared to the control group; adjusted association: aRR = 4.23, 95% CI 3.46–5.17 for siblings and aRR = 5.07, 95% CI 4.13–6.23 for patients). The unadjusted RR was significantly greater for patients than for siblings (χ^2^(1) = 18.12, *p* < 0.001); and the same was the case for the adjusted RR (χ^2^(1) = 3.89, *p* = 0.049) .

**Table 3.** JTC bias per group

		Controls (*n* = 1513)	Siblings (*n* = 1185)	Patients (*n* = 1055)	Total (*n* = 3753)
JTC bias					
	No	1260 (83%)	674 (57%)	505 (48%)	2439 (65%)
	Yes	253 (17%)	511 (43%)	550 (52%)	1314 (35%)

**Table 4.** Results (RR and 95% CI) on the association between JTC bias and psychosis risk outcome

	Psychosis risk = 0 (controls)	Psychosis risk = 1 (siblings)		Psychosis risk = 2 (patients)		Difference in RR (patients *v.* siblings)
	Reference group	RR (95% CI)	*p*	RR (95% CI)	*p*	χ^2^
Reasoning bias:						
Unadjusted model	–	3.78 (3.16–4.51)	<0.001	5.42 (4.53–6.50)	<0.001	χ^2^ = 18.12, df = 1, p < 0.001
Adjusted model^a^	–	4.23 (3.46–5.17)	<0.001	5.07 (4.13–6.23)	<0.001	χ^2^ = 3.89, df = 1, *p* = 0.049

*Note*: CI = confidence interval, RR = relative risk ratio.

a Model adjusted for socio-demographics (age, sex, level of education, cognitive score, country and being a member of the same family).

### Interaction between JTC bias and delusions

Multinomial logistic regression analysis showed a significant interaction between JTC bias and CAPE delusions, with an adjusted interaction term of 3.77 (95% CI 1.67–8.51) in the sibling-control model and a similarly directed but statistically imprecise aRR of 2.15 (95% CI 0.93–4.92) in the patient-control model (difference in interaction term siblings *v.* patients: χ^2^ = 3.06, df = 1, *p* = 0.08) (Table 5). The interaction between JTC and CAPE visual hallucinations was in the same direction but non-significant (aRR siblings: 2.28, 95% CI 0.71–7.35 and aRR patients: 1.39, 95% CI 0.58–3.35), with similar results for the interaction between JTC and CAPE auditory hallucinations (sibling group aRR: 1.63, 95% CI 0.37–7.08 and patient group aRR: 1.51, 95% CI 0.41–5.55).

**Table 5.** Results (RR and 95% CI) on the interaction between lifetime positive psychotic experiences and JTC bias on psychosis risk outcome

	Psychosis risk = 0 (controls) Reference group	Psychosis risk= 1 (siblings)^a^		Psychosis risk = 2 (patients)^a^	
		RR (95% CI)	*p*	RR (95% CI)	*p*
Interaction of lifetime psychotic experiences and reasoning bias:					
Delusions × JTC bias	–	3.77 (1.67–8.51)	0.001^b^	2.15 (0.94–4.92)	0.071^b^
Visual hallucinations × JTC bias	–	2.28 (0.71–7.35)	0.166	1.39 (0.58–3.35)	0.465
Auditory hallucinations × JTC bias	–	1.63 (0.37–7.08)	0.517	1.51 (0.41–5.55)	0.535

*Note*: CI = confidence interval, RR = relative risk ratio.

a Model adjusted for socio-demographics (age, sex, level of education, cognitive score, country and being a member of the same family).

b Difference in interaction term siblings *v.* patients: χ^2^ = 3.06, df = 1, *p* = 0.08.

## Discussion

The results show that patients with psychotic disorders, as well as their non-ill siblings, were more likely than controls to show a JTC bias. The association was furthermore contingent on evidence of delusional ideation, JTC being most strongly associated with sibling status if there was also evidence of delusional ideation. No such association was found for visual or auditory hallucinations. The results replicate earlier findings from the study by Van Dael et al. (2006), in which JTC bias was similarly found to be associated with psychosis liability (i.e. patients and relatives showing the highest probability of JTC bias compared to healthy controls), and in which a similar interaction was observed between psychosis liability and evidence of delusional ideation in the model of JTC (Van Dael et al., 2006).

### Methodological considerations

A strength of this study is that we followed a rigorous method, and strictly conformed to the procedure and sampling of the original study to achieve a true replication in a much larger and independent study population. Nevertheless, there are several methodological limitations that need attention. First, we investigated lifetime psychosis expression; and therefore, an inference based on the differentiation between lifetime and current symptomatology might be somewhat difficult to draw. However, the finding that non-ill first-degree relatives show greater JTC bias than controls, adds to the assumption that JTC bias may indeed represent a trait characteristic of psychosis. The study sample largely consisted of patients with an established diagnosis over a period longer than 5 years (average duration of illness since the age of the first contact with mental health services: 9.9 years); and therefore, our findings may not be entirely generalizable to earlier stages of psychotic illness. Second, the CAPE is developed to investigate psychotic experiences in the general population; therefore, ceiling effects might have occurred in the patient group. However, we should note that this would have caused an underestimation rather than an overestimation of the moderating effect of delusional ideation on the association between JTC bias and patient status. As patients are likely to experience more severe symptoms than assessed by CAPE self-report, this could also explain why the interaction term for the patients was imprecise. Third, we did not include specific cognitive measures to investigate whether JTC bias is a specific cognitive deficit or whether it reflects a more general cognitive impairment. Tripoli et al. (2020) investigated associations between JTC bias, psychosis and general cognition and found that case-control differences in JTC were mediated by IQ. The authors conclude that JTC bias may be a manifestation of a more general cognitive impairment rather than being a specific cognitive deficit associated with psychosis status. Although we did not calculate mediation effects, we adjusted for general cognition score and still found JTC bias to be significantly associated with the degree of psychosis liability. More research, however, is needed to clarify the specificity of the association between JTC bias (as opposed to other cognitive biases and general cognition score) and psychosis.

### JTC and delusions

The results of this study suggest that the association between JTC and psychosis liability is specific for delusions (and not hallucinations). This was found not only in patients with an established diagnosis of schizophrenia spectrum disorder but also in their first-degree relatives. Freeman similarly described such an association between JTC bias and delusion proneness in non-clinical individuals using a virtual reality experiment (Freeman, Pugh, Vorontsova, Antley, & Slater, 2010). So and Kwok (2015) as well as Warman, Lysaker, Martin, Davis, and Haudenscheid (2007) however, did find evidence for an association between JTC and delusions in patients but not for JTC bias and delusion proneness in non-clinical samples. A recent meta-analysis concluded that JTC does covary with delusions across diagnoses and that JTC is not just associated with mental disorder in general. The association with delusions however, might be more specifically present at the more severe end of the continuum of delusional beliefs (McLean et al., 2017).

Longitudinal studies that examine the temporal dynamics of JTC and delusions are limited. To our knowledge, no study to date has investigated whether JTC actually precedes the formation of delusions in healthy controls or individuals with above-average psychosis liability. Experimental studies manipulating JTC and examining the effects thereof on delusion severity have yielded mixed results thus far (Garety et al., 2015; Moritz, Veckenstedt, Randjbar, Vitzthum, & Woodward, 2011; Ross, Freeman, Dunn, & Garety, 2011; Schneider et al., 2016).

### Is JTC an endophenotype for psychosis?

Van Dael et al. (2006) found that JTC bias is present not only in patients with an established diagnosis of psychosis spectrum disorder but also in those with above-average (familial) liability for psychosis. The current analyses in a larger, multi-site sample confirm previous findings by showing that also unaffected family members have a higher degree of JTC bias than the general population. More evidence in support of the notion that JTC bias might be an endophenotype for psychosis comes from studies investigating JTC bias in the general population. These general population studies have revealed that JTC is more frequently present in individuals with prodromal symptoms of psychosis (Broome et al., 2007) as well as in individuals scoring high on the CAPE (Lincoln, Lange, Burau, Exner, & Moritz, 2010) and other measures of subclinical delusional ideation (Menon et al., 2013). Recently, JTC was found to be associated with co-occurring psychotic experiences and affective disturbances in a general population sample (but not with the sole presence of psychotic experiences nor affective disturbances), which suggests that JTC might contribute to the transdiagnostic phenotype of co-occurring psychotic and affective symptoms (Reininghaus et al., 2019). Ludtke, Kriston, Schroder, Lincoln, and Moritz (2017) investigated fluctuations in JTC bias over time using Experience Sampling Methodology and found JTC bias to be stably present in daily life, while fluctuations in JTC over the course of days also occurred. Moreover, fluctuations in JTC magnitude co-varied with psychosis expression. In conclusion, these findings replicate earlier findings that JTC bias is associated with familial liability for psychosis and that this is contingent on the degree of delusional ideation. Future studies are required to investigate the JTC bias longitudinally across the severity, continuity, and course of psychosis spectrum: acute *v.* remission, recent onset *v.* later stage and relatives *v.* controls.
